# Treatment and outcomes of hepatoblastoma in a tertiary care pediatric institution: a single-center experience

**DOI:** 10.3389/fonc.2026.1943323

**Published:** 2026-09-03

**Authors:** Aleksandar Sretenović, Srđa Janković, Nada Krstovski, Polina Pavićević, Jelena Lazić, Goran Milošević, Marija Ćazić, Jelena Krcunović, Branislav Jovanović, Tijana Radović, Đorđe Pavlović, Dubravka Milutinović, Jelena Pejanović Jovanović, Ivana Dašić, Sofija Cvejić, Jovana Svorcan Pavlović, Predrag Rodić

**Affiliations:** 1 Faculty of Medicine, University of Belgrade, Belgrade, Serbia; 2 Department of Abdominal Surgery, University Children’s Hospital, Belgrade, Serbia; 3 Department of Immunology, University Children’s Hospital, Belgrade, Serbia; 4 Department of Hematology And Oncology, University Children’s Hospital, Belgrade, Serbia; 5 Department of Radiology, University Children’s Hospital, Belgrade, Serbia

**Keywords:** hepatoblastoma, liver resection, pediatric liver tumors, pretext, treatment outcome

## Abstract

**Introduction:**

Hepatoblastoma is the most prevalent pediatric malignant liver neoplasm, and despite substantial advances in multimodal treatment, outcome data from Southeast European pediatric centers remain limited. The aim of this study was to evaluate the clinical characteristics, treatment approaches, surgical management, and outcomes of children treated for hepatoblastoma at a tertiary pediatric institution over a 17-year period.

**Methods:**

This retrospective single-center cohort study included 24 pediatric patients treated between January 2008 and March 2025. Clinical variables included age, sex, tumor size, PRETEXT stage, metastatic disease, and vascular involvement. Treatment variables comprised chemotherapy protocol, surgical procedure, liver transplantation, and resection margin status, while outcome measures included relapse, overall survival, and normalization of serum α-fetoprotein levels.

**Results:**

With the exception of one child who died before treatment could be initiated, all patients underwent surgical treatment following liver biopsy and preoperative chemotherapy. Overall, six children (25.0%) died, whereas 18 were alive at the time of analysis, corresponding to an overall survival rate of 75.0% with a median follow-up of 9.0 years. All patients with PRETEXT 1 and PRETEXT 2 disease survived, whereas survival among patients with PRETEXT 3–4 disease was significantly lower (100.0% vs. 50.0%, p = 0.014). Postoperative relapse occurred in two of the 23 treated patients (8.7%), and serum α-fetoprotein levels normalized after treatment in all surviving patients.

**Discussion:**

In this retrospective single-center cohort, multimodal treatment of hepatoblastoma achieved an overall survival of 75.0%, with particularly favorable outcomes in patients with PRETEXT 1–2 disease and negative surgical margins. These findings provide valuable long-term real-world evidence on hepatoblastoma management at a tertiary pediatric referral center.

## 1 Introduction

Hepatoblastoma is the most prevalent pediatric malignant neoplasm of the liver, comprising about 1% of all childhood cancers, with an estimated incidence of 1.5 cases per million children per year (1). It occurs predominantly in very young children, with only 5% of cases presenting after the age of four. With the advent of modern disease staging systems and treatment protocols, the overall survival of children with hepatoblastoma has markedly improved (2). Indeed, the global number of deaths attributable to this tumor has been approximately halved over the past three decades (3). However, up to 20% of patients still have a relatively unfavorable prognosis due to risk factors such as metastatic disease at presentation, multifocal tumor origin, vascular invasion, or extrahepatic spread. Although the burden of hepatoblastoma shows a downward trend in some countries and/or regions, with a concomitant improvement of patient survival, increases in incidence and mortality still occur in many areas with unfavorable socioeconomic conditions (4).

Hepatoblastoma patients are routinely classified according to the Pretreatment Extent of Disease (PRETEXT) system, comprising a score determined by abdominal computed tomography (CT) scanning and defined by the International Society of Pediatric Oncology Liver Tumor Study Group (SIOPEL) (5, 6). This classification is primarily based on the number of involved Couinaud liver segments, in addition to the presence or absence of extrahepatic tumor extension, involvement of inferior vena cava or hepatic veins, and metastatic disease at the time of diagnosis. Therefore, a thorough radiological assessment is essential for accurate tumor staging. Serum α-fetoprotein levels and their dynamics during treatment are important diagnostic parameters in hepatoblastoma (5). The principal treatment modality for hepatoblastoma is complete surgical resection, which is typically preceded by neoadjuvant chemotherapy. Liver transplantation is generally reserved for patients in whom complete resection is not feasible and may represent a life-saving therapeutic intervention (7). Complete tumor resection remains one of the most critical determinants of a favorable clinical outcome.

Despite substantial advances in multimodal treatment, outcome data from Southeast European pediatric centers remain limited. The aim of the present study was to evaluate the clinical characteristics, treatment approaches, surgical management, and outcomes of children treated for hepatoblastoma at a tertiary pediatric institution over a 17-year period.

## 2 Materials and methods

This retrospective single-center cohort study included all pediatric patients treated for hepatoblastoma at the University Children’s Hospital, Belgrade, between January 2008 and March 2025. We reviewed the medical records of 24 patients (13 [54.2%] male, 11 [45.8%] female, median age 1.0 years [range 0.1-9.4 years]). Clinical variables retrieved included age, sex, tumor size, PRETEXT stage (acquired by using CT or MRI angiography), metastatic disease and vascular involvement. Treatment variables included chemotherapy protocol, operative procedure, transplantation and margin status, while outcome variables comprised occurrence of relapse, patient survival and α-fetoprotein (AFP) normalization. Treatment protocols evolved during the study period in accordance with contemporary international SIOPEL and PHITT recommendations. SIOPEL-3 treatment consisted of either cisplatin (CDDP, 80 mg/m^2^ every 14 days; six courses) or PLADO (CDDP 80 mg/m^2^ plus doxorubicin 60 mg/m^2^ per cycle). SIOPEL-6 consisted of six courses of CDDP 80 mg/m^2^ every 14 days (four preoperative and two postoperative); sodium thiosulfate was not used in our cohort. PHITT Group B patients received CDDP 80 mg/m^2^ every 14 days (4–6 courses), Group C patients were treated according to the SIOPEL-3HR arm (alternating CDDP 80 mg/m^2^ and carboplatin 500 mg/m^2^ plus doxorubicin 60 mg/m^2^), while Group D patients received SIOPEL-4-based induction followed, after complete metastatic response and surgery, by three cycles of carboplatin 500 mg/m^2^ plus doxorubicin 40 mg/m^2^. Complete dosing schedules and protocol-specific modifications are provided in the original SIOPEL and PHITT publications. Continuous variables were expressed as median, range, and standard deviation (SD), while categorical variables were summarized as frequencies and percentages. Given the small sample size and sparse cell counts, comparisons between categorical variables were performed using Fisher’s exact probability test. A p value <0.05 was considered statistically significant.

## 3 Results

### 3.1 Tumor characteristics

The median longest tumor diameter was 10.7 cm (SD 2.7 cm, range 8.0-18.8 cm). In five children (20.8%), the tumor was confined to the left hepatic lobe, while 11 (45.8%) had tumors confined to the right lobe, and eight (33.3%) had bilobar involvement. Tumor size, affected liver segments, PRETEXT stage and patient demographic characteristics are summarized in **Table 1**. Metastatic disease at presentation was observed in one patient (4.2%). Tumor invasion of the inferior vena cava was identified in five patients (20.8%), whereas portal vein involvement was present in one patient (4.2%). The median AFP level was 101,940 ng/mL (range 816-2,148,540 ng/mL). Serum AFP levels normalized following the last postoperative cycle of chemotherapy in all survived patients.

**Table 1:** Demographic, radiologic, and staging characteristics of patients with hepatoblastoma.

Pt. no.	Age (y)	Sex	Ø (cm)	Lobe(s) affected	Segment(s) affected	PRETEXT	Metastatic disease at presentation
1	0.6	M	9.0	R	3,5,6,7,8	4	No
2	1.0	F	11.6	R	5,6,7,8	2	No
3	0.5	F	12.5	L,R	4,5,6,7	3	No
4	1.9	M	18.8	R	3,5,6,7,8	3	No
5	0.1	F	9.0	L	2,3	1	No
6	1.3	F	14.5	L,R	4,5,6,7,8	3	No
7	0.9	F	10.0	R	4,5,6,7,8	3	No
8	9.4	F	14.5	L,R	4,5,6,7,8	3	No
9	7.3	M	16.0	L	2,3,4,5,8	3	No
10	1.2	F	9.2	R	5,6,7,8	2	No
11	0.9	M	10.7	R	5,6	2	No
12	0.7	M	11.5	R	6,7	1	No
13	1.0	M	11.0	L,R	4,5,8	3	No
14	1.9	F	8.0	L	2,3,4	2	No
15	1.9	M	8.4	R	5,7,8	2	No
16	1.2	M	10.3	L,R	2,5,6,7,8,E	4	No
17	0.2	M	10.6	L,R	4,5,8	3	No
18	0.6	F	14.0	L,R	All	4	Yes
19	0.1	M	13.3	L	2,3,4b	2	No
20	2.2	F	8.7	R	8,5	2	No
21	3.8	M	9.7	R	6,7	2	No
22	4.8	M	14.1	L,R,PV	2,5,6,7	4	No
23	0.7	M	10.5	L	2,3,4	2	No
24	0.7	F	9.0	R	6,7	2	No

E, extrahepatic involvement; F, female; L, left lobe; M, male; PRETEXT, Pretreatment Extent of Tumor; PV, portal vein; R, right lobe; Ø, longest tumor diameter.

Histopathological examination demonstrated epithelial-type hepatoblastoma in 12 patients (50.0%) and mixed epithelial-mesenchymal hepatoblastoma in 11 (45.8%). Among the 12 epithelial tumors, six (50.0%) were of the fetal subtype, two (16.7%) were embryonal, one (8.3%) was mixed embryonal-fetal, and further classified was not done in three (25%) patients. In one patient (4.2%) tumor biopsy and the histopathological assessment could not be performed due to early death upon referral (**Table 2**).

**Table 2:** Biological, vascular and histopathological characteristics of the tumors.

Pt. No.	AFP (ng/mL)	AFP normalization	Inf. vena cava infiltration	Pathohistological type
1	160003	No	No	Epithelial/mesenchymal
2	18513	Yes	No	Epithelial/mesenchymal
3	5845	Yes	No	Epithelial, embryonal
4	485262	No	Yes	NA
5	104940	Yes	No	Epithelial/mesenchymal
6	343455	Yes	Yes	Epithelial/mesenchymal
7	36300	No	Yes	Epithelial, fetal
8	206203	Yes	No	Epithelial/mesenchymal
9	121000	Yes	Yes	Epithelial, unspecified
10	435106	Yes	Yes	Epithelial, embryonal/fetal
11	363363	Yes	No	Epithelial/mesenchymal
12	87161	Yes	No	Epithelial, fetal
13	98940	Yes	No	Epithelial/mesenchymal
14	60500	Yes	No	Epithelial/mesenchymal
15	10000	Yes	No	Epithelial, fetal
16	10000	Yes	No	Epithelial, fetal
17	212982	No	No	Epithelial, fetal
18	16600	Yes	No	Epithelial, fetal
19	1790000	Yes	No	Epithelial, embryonal/fetal
20	79206	Yes	No	Epithelial/mesenchymal
21	816	Yes	No	Epithelial, fetal
22	300000	Yes	No	Epithelial, embryonal
23	2148540	Yes	No	Epithelial/mesenchymal
24	60000	Yes	No	Epithelial, unspecified

AFP, α-fetoprotein; NA, not applicable.

### 3.2 Treatment and outcome

With the exception of one child who died before treatment could be initiated, all patients underwent surgical treatment following liver biopsy and preoperative chemotherapy. One child (4.3%) underwent liver transplantation and achieved long-term survival, whereas the remaining patients underwent surgical resection. An open surgical approach was used in all 22 patients, without the use of intraoperative ultrasonography. All hepatic resections were performed using Cavitron Ultrasonic Surgical Aspirator (CUSA; Valley lab, Boulder, CO, USA). Of the 22 patients, 9 (40.9%) underwent segmental liver resection, 8 (36.4%) underwent hepatic lobectomy (7 right-sided [31.8%] and 1 left-sided [4.5%]), 3 (13.6%) underwent mesohepatectomy, and 2 (9.1%) underwent extended hepatectomy. Postoperative complications occurred in 3 of 22 patients (13.6%). Two patients (9.1%) developed surgical site infections, classified as Clavien-Dindo grade II, which were successfully treated with antibiotics and daily wound dressing. One patient (4.5%) developed a small pleural effusion (Clavien-Dindo grade I), which resolved spontaneously. The preoperative treatment protocols and surgical procedures performed are presented in **Table 3**. Of the five patients treated according to SIOPEL 3 regimens, two survived, whereas all nine patients treated according to SIOPEL 6 were alive at the time of analysis. Among the nine patients subsequently treated according to PHITT-based risk-adapted strategies, seven survived. Within the latter group, all five low-risk group B patients survived, compared with one of two intermediate-risk group C patients and one of two high-risk group D patients. One additional patient died before treatment could be initiated. Given the very small and clinically heterogeneous subgroups, no formal statistical comparisons between treatment protocols or eras were performed.

**Table 3:** Treatment protocols, surgical management and outcomes.

Pt. No.	Tumor biopsy	Treatment protocol	Tumor reduction	Resected Lobe(s)/segment(s)	Tumor present at resection margin	Vital outcome
1	Yes	SIOPEL 3, PLADO	Yes	RL	Yes	Deceased
2	Yes	SIOPEL 3, PLADO	Yes	S5,S6,S7	No	Alive
3	Yes	SIOPEL 3, CDDP	Yes	S4,S5,S6	No	Alive
4	No	N.p.	N.a.	NP	NA	Deceased
5	Yes	SIOPEL 6, CDDP	Yes	S2,S3	No	Alive
6	Yes	SIOPEL 3, PLADO	Yes	RL,S4	No	Deceased
7	Yes	SIOPEL 6, CDDP	Yes	RL	No	Alive
8	Yes	SIOPEL 3, PLADO	Yes	RL,S4	Yes	Relapsed and Deceased
9	Yes	SIOPEL 6, CDDP	Yes	LL	No	Alive
10	Yes	SIOPEL 6, CDDP	Yes	RL	No	Alive
11	Yes	SIOPEL 6, CDDP	Yes	S5,S6	No	Alive
12	Yes	SIOPEL 6, CDDP	Yes	S6,S7	Yes	Alive
13	Yes	SIOPEL 6, CDDP	Yes	S4,S8	No	Alive
14	Yes	SIOPEL 6, CDDP	Yes	RL,CL	Yes	Alive
15	Yes	SIOPEL 6, CDDP	Yes	RL	No	Alive
16	Yes	PHITT, gr. C, IR	Yes	S2,S3,S4a,S4b,S5,S6,S7	No	Alive
17	Yes	PHITT, gr. C, IR	Yes	S4a,S4b,S5,S7	No	Deceased
18	Yes	SIOPEL 4, PHITT, gr. D, HR	Yes	LT	NA	Alive
19	Yes	PHITT, gr. B, LR	Yes	S2,S3	No	Alive
20	Yes	PHITT, gr. B, LR	Yes	S5,S8	No	Alive
21	Yes	PHITT, gr. B, LR	Yes	S6,S7	No	Alive
22	Yes	PHITT, gr. D, HR	Yes	S6,S7	Yes	Relapsed and Deceased
23	Yes	PHITT, gr. B, LR	Yes	S2,S3	No	Alive
24	Yes	PHITT, gr. B, LR	Yes	S6,S7	No	Alive

CDDP, cis-diamminedichloroplatinum (cisplatin); CL, caudate lobe; HR, high risk; IR, intermediate risk; LL, left lobe; LR, low risk; LT, liver transplantation; NA, not applicable; NP, not performed; PHITT, Pediatric Hepatic International Tumor Trial; PLADO, chemotherapy regimen combining cisplatin and doxorubicin; S, segment; RL, right lobe.

All treated patients demonstrated a reduction in tumor size following preoperative chemotherapy. Postoperative relapse occurred in two of the 23 treated patients (8.7%). Among the 22 patients who underwent hepatic resection, surgical margins were tumor-free in 17 (77.3%) whereas microscopic residual disease was present in five (22.7%). Of the 17 patients with negative resection margins, 15 (88.2%) survived, compared to two of five patients (40.0%) with positive margins – a marginally non-significant difference (p=0.055). All patients with PRETEXT 1 (2/2) and all 10 with PRETEXT 2 disease (10/10) survived. Survival was observed in four of eight patients (50.0%) with PRETEXT 3 disease and two of four patients (50.0%) with PRETEXT 4 disease. Given the very small number of patients within individual PRETEXT categories, particularly PRETEXT 1 and PRETEXT 4, formal comparisons across all four groups would have been statistically unreliable. We therefore performed an additional exploratory comparison of aggregated PRETEXT 1–2 versus PRETEXT 3–4 groups, while retaining the individual-stage results above. This dichotomization should be interpreted as a sample-size-driven exploratory analysis and does not imply prognostic equivalence between PRETEXT 1 and 2 or between PRETEXT 3 and 4. Survival was 12/12 (100.0%) in the PRETEXT 1–2 group and 6/12 (50.0%) in the PRETEXT 3–4 group (p=0.014). Given the small subgroup sizes, this finding should be interpreted cautiously. Overall survival was 83.3% (10/12) among patients with epithelial hepatoblastoma and 63.6% (7/11) among those with mixed epithelial/mesenchymal type. This difference was not statistically significant (p=0.371). However, due to the same sample size limitation, this non-significance might in fact reflect a type II inferential error rather than a true lack of difference. Differential survival between these subgroups is shown in **Table 4** and the Kaplan-Meier diagram of patient survival in (**Supplementary Figure 1**).

**Table 4:** Patient survival according to PRETEXT, histopathological type and margins status.

Variable	Subgroup	Survival	p-value
PRETEXT	PRETEXT 1-2	12/12 (100.0%)	0.014*
	PRETEXT 3-4	6/12 (50.0%)	
HP type	Epithelial	10/12 (83.3%)	0.371
	Mixed Epitelial/Mesenchymal	7/11 (63.6%)	
Margins	Negative	15/17 (88.2%)	0.055
	Positive	2/5 (40.0%)	

*p < 0.05. HP, histopathological.

Overall, six children (25.0%) died, while 18 were still alive at the time of analysis, corresponding to an overall survival of 75.0% and a median follow-up of 9.0 years (range 1.5-17.2 years). In addition to the child who died before treatment could be administered (patient #4) and the patient who died intraoperatively (patient #1), two patients died following relapse and disease progression (patients #8 and patient #22). Patient #4 was referred urgently from a remote region of the country and admitted through the emergency surgical service with a large hepatic tumor and clinical signs of intratumoral hemorrhage. Initial diagnostic evaluation included abdominal CT and serum AFP measurement. Despite urgent assessment, progressive intratumoral bleeding led to hemorrhagic shock, and the child died before definitive surgical management or oncological treatment could be initiated. Patient #8 developed an early combined local and metastatic relapse and received salvage chemotherapy with etoposide and carboplatin. The disease was refractory to treatment, and the patient subsequently received palliative care and died of progressive disease. Patient #22 developed a local relapse and received one course of etoposide/carboplatin followed by two courses of irinotecan/vincristine. He was subsequently referred abroad for liver transplantation. Despite transplantation, the disease relapsed again and the patient ultimately died of progressive disease. The remaining deaths resulted from postoperative hemorrhage (patient #6) and hepatic failure complicated by hepatorenal syndrome (patient #17), respectively. Thus, of the six fatal outcomes, two were associated with progressive disease or relapse, while four resulted from perioperative or postoperative complications. Among the six patients who died, two were classified as having PRETEXT 4 disease, and four as PRETEXT 3 disease (**Table 5**). Of the two patients with epithelial hepatoblastoma who died, one had the fetal subtype and the other had the embryonal subtype.

**Table 5:** Causes of death.

Pt. no.	PRETEXT	Time to death (y)	Circumstances/proximate cause of death
1	4	0.5	Died intraoperatively
4	3	0.0	Died at initial evaluation
6	3	0.7	Died of postoperative bleeding
8	3	0.9	Died upon relapse
17	3	0.6	Died of liver insufficiency
22	4	2.8	Died upon relapse and liver transplant

y, year(s).

## 4 Discussion

In this retrospective single-center series, multimodal treatment of hepatoblastoma resulted in a 75% overall survival, with particularly favorable outcomes in patients with PRETEXT 1–2 disease and negative surgical margins. Treatment is conducted according to current international protocols. However, formal participation in European cooperative treatment frameworks remains limited because Serbia is not currently a member of the European Union. Nevertheless, our national group officially participated in SIOPEL 3 and continues to follow up-to-date European protocols; however, patients in the present series were not subjected to treatment randomization as per protocol but were assigned to treatment arms based on treating clinician’s experience-guided decisions. The present study has several limitations, including its retrospective single-center design, relatively small sample size, and heterogeneity of treatment protocols over the long study period. In addition, the limited number of data points precluded a multivariant statistical analysis. Nevertheless, the study may provide valuable long-term real-world data regarding hepatoblastoma management in a tertiary pediatric referral center.

The overall survival in our patient series (18/24, 75.0%) roughly corresponds to outcomes reported in the literature. For instance, Koh et al. reported a five-year overall survival of 80.2% across all risk groups, while Shah et al. reported 81.8% and Tang et al. as much as 93.5% (8–10).

On the other hand, a series of 22 patients with high-risk hepatoblastoma from Chennai (India) featured a five-year overall survival of 62% (11). In a published 20-year experience from Turkiye, the five-year overall survival was 80.8% (12). Quantitative comparison of these results to our series is, however, slightly hampered by the wide variation in the follow-up duration in our patients. We did find the outcome of hepatoblastoma treatment in the University Children’s Hospital has been improved with respect to our own previously published results (13). Although what is achieved by current treatment protocols can hardly be overstated, a recent survey of 52 surgeons versed in hepatoblastoma treatment, including 28 who routinely perform liver transplantation, demonstrated that there is still work to be done regarding the standardization and unification of the surgical approach (14). All patients in our series, prior to surgery, underwent a biopsy with pathohistological evaluation, which is an important step toward such standardization compared to the rather inconsistent practice in the preceding period. In this context, the fact that all patients showed a tumor reduction after preoperative chemotherapy is very significant, and is in line with the worldwide published experience. The latter particularly underscores the importance of the first two hemiotherapeutic cycles (15).

Tumors in the present series were, in general, relatively large at presentation, their median longest diameter exceeding 10 cm. While this begs the question whether, or how much, could still be gained by improving the timeliness of diagnosis, it should also be born in mind that hepatoblastoma is a swiftly growing tumor affecting an organ produces symptoms only after a significant damage (and corresponding humor growth) has occurred. Patient #4, who died during the initial evaluation, was referred urgently from a geographically remote region with a large hepatic tumor complicated by intratumoral hemorrhage. Despite immediate diagnostic assessment after admission, progressive bleeding resulted in hemorrhagic shock before definitive surgical or oncological treatment could be initiated. Although the contribution of delayed healthcare access cannot be quantified in an individual case, the advanced presentation and emergency referral from a remote area highlight the potential importance of timely recognition and referral of children with suspected hepatic tumors. This case therefore illustrates how geographic and healthcare-access disparities may compound the clinical challenges posed by the rapid growth and occasionally acute presentation of hepatoblastoma. Apart from highlighting the significance of early diagnosis, this episode attests that there is much room for improvement in early diagnostic capabilities of our primary healthcare institutional network, resonating with the United Nations-stipulated Millenium Development Goal to “leave no-one behind”. For context, in a recently published series from Pakistan, as much as 53% of children with hepatoblastoma presented with a tumor that was no longer operable (16). In a similar vein, a Brazilian study on mortality trends in hepatoblastoma comprehensively demonstrated a substantial influence of socioeconomic disparities and regionally heterogenous organization of the healthcare system on hepatoblastoma outcomes (17).

The only child in our series that was treated by a liver transplant (patient #18) survived. Of the two patients who relapsed, patient #8 developed rapidly progressive local and metastatic disease that was refractory to salvage chemotherapy, whereas patient #22 underwent liver transplantation after salvage chemotherapy for local relapse but subsequently developed a further recurrence and died. These cases illustrate the particularly challenging management of relapsed hepatoblastoma and the limited curative options once disease recurs despite multimodal salvage treatment. Additionally, there is a large debate as to whether some hepatoblastoma patients without signs of metastatic disease at presentation should nevertheless be treated by transplantation and, if so, what exact criteria should be applied to opt for such treatment (18). An important (and quite common) constraint in this decision is the availability of a matched related liver donor. Our institution currently has no access to an affiliated transplantation center, nor a possibility of cadaveric liver transplantation. Consequently, our patient was transplanted abroad. Another child with complex tumor spread (patient #16) was successfully treated in a foreign center without liver transplantation.

Of the five children with inferior vena cava infiltration, two died (patients #4 and #6) while three survived (patients #7, #9 and #10). While the sample is clearly insufficient to allow any conclusions, it is still a good reminder that caval infiltration, although an unfavorable prognostic factor and an additional surgical challenge, may not in itself be an ominous sign (19). The assessment of potential impact of caval involvement is complicated by the difficulty to radiographically distinguish true infiltration from mere displacement of the vena cava by the tumor mass. The same is true of the hepatic vein. Thus, it is possible (although beyond any possibility of verification) that some of the four children who died and in whom no caval infiltration was noted might have had, in fact, a non-detected microscopic infiltration adversely affecting the overall outcome. It is also notable that the single child with an infiltration of the portal vein relapsed as well.

Five patients in our series showed a visible presence of tumor at resection margins: three died and two are alive at the time of writing. Although this difference did not reach statistical significance (p=0.055), the absolute difference in observed survival between patients with negative and positive resection margins was 48.2 percentage points (88.2% vs. 40.0%). Given the very small number of patients with positive margins, this finding should not be interpreted as evidence of an independent prognostic effect; nevertheless, its magnitude may be clinically meaningful and warrants cautious interpretation. Infiltrated margins obviously exert an impact on prognosis, but the latter appears rather difficult to quantify, since published reports vary widely in this regard, with the effect of marginal infiltration on patient survival ranging from almost negligible as reported by Ren et al. to quite considerable as reported by Weeda et al. (20, 21). A recent meta-analysis from Heidelberg found no adverse oncological consequences of microscopically infiltrated resection margins; however, due to heterogeneity of available data and other limitations, no firm conclusion can be drawn at this time and tumor-free margins justifiably remain a strong desideratum (22).

Last but not least, all children with PRETEXT 1 and 2 are alive and well at the time of writing, while overall survival in those with PRETEXT 3 and 4 was 50.0% (4/8 and 2/4, respectively). This does not deviate significantly from the expected distribution. However, our results are in line with the observation that time elapsed before diagnosis as well as total tumor volume at presentation may be more important than PRETEXT per se, particularly in resource-poor settings (23). It should also be noted that, particularly given the existence of many confounding variables that our study was hopelessly underpowered to isolate, PRETEXT may not be an independent predictor of outcome but a surrogate indicator of primary predictors.

The improvement observed compared with our previously published results coincided with the period during which almost all hepatoblastoma procedures were performed by a single surgeon, allowing the concentration of surgical experience in the management of this rare disease. Since then, perioperative mortality has been significantly reduced. Notably, the only child who died intraoperatively (patient #1) was one of four patients in our series who were not operated on by the designated hepatoblastoma surgeon. However, this temporal association cannot establish causality, since the same period was also characterized by evolution of chemotherapy protocols, improvements in imaging and perioperative management, and advances in supportive care. The improved outcomes should therefore be interpreted as reflecting the cumulative evolution of multidisciplinary care rather than the independent effect of any single component. The above mentioned findings underscore the importance of centralizing hepatoblastoma management within experienced multidisciplinary teams, particularly with regard to the assessment of tumor resectability, as also emphasized by Uchida et al. (24). This issue is all the more important since liver tumors (and, by extension, liver resections) are exceedingly rare in childhood, and direct transfer of experiences from adult liver surgery is not possible (25). Recent reports further illustrate that hepatoblastoma management may require considerable individualization in uncommon clinical settings, including primary surgical resection of localized disease in an adolescent and neoadjuvant cisplatin-based treatment followed by resection in congenital hepatoblastoma (26, 27).

In addition to further refinements in protocol and technique, the future of hepatoblastoma treatment may also entail robotic surgery and an increasing reliance on artificial intelligence (28, 29). Upcoming treatment protocols could also benefit from emerging insights into hepatoblastoma biology and cellular diversity, with a plethora of newly discovered tumor-initiating and tumor-driving mechanisms, as well as a rising understanding of key roles of the tumor microenvironment in its progression (30). Further improvements in risk stratification and a protocol selection tailored to all available knowledge will most likely translate into still better patient outcomes, and, in time, these advances may further improve survival while reducing treatment-related morbidity (31).

## 5 Conclusion

While our single-center experience demonstrates a substantial improvement in overall treatment outcomes for hepatoblastoma compared with previous results, there remains a considerable scope for further progress, particularly with regard to early diagnosis, risk-adapted treatment strategies and decision-making concerning the use of liver transplantation. Centralization of care within an experienced multidisciplinary team appears to play an important role in optimizing outcomes.

## Data Availability

The raw data supporting the conclusions of this article will be made available by the authors, without undue reservation.
