# Supplementary material for: Treatment and outcomes of hepatoblastoma in a tertiary care pediatric institution: a single-center experience
Source: Front Oncol. 2026 Sep 3;16:1943323. doi: 10.3389/fonc.2026.1943323 (PMC13581549; doi:10.3389/fonc.2026.1943323)

Supplementary Figure 1. Kaplan-Meier survival diagram.

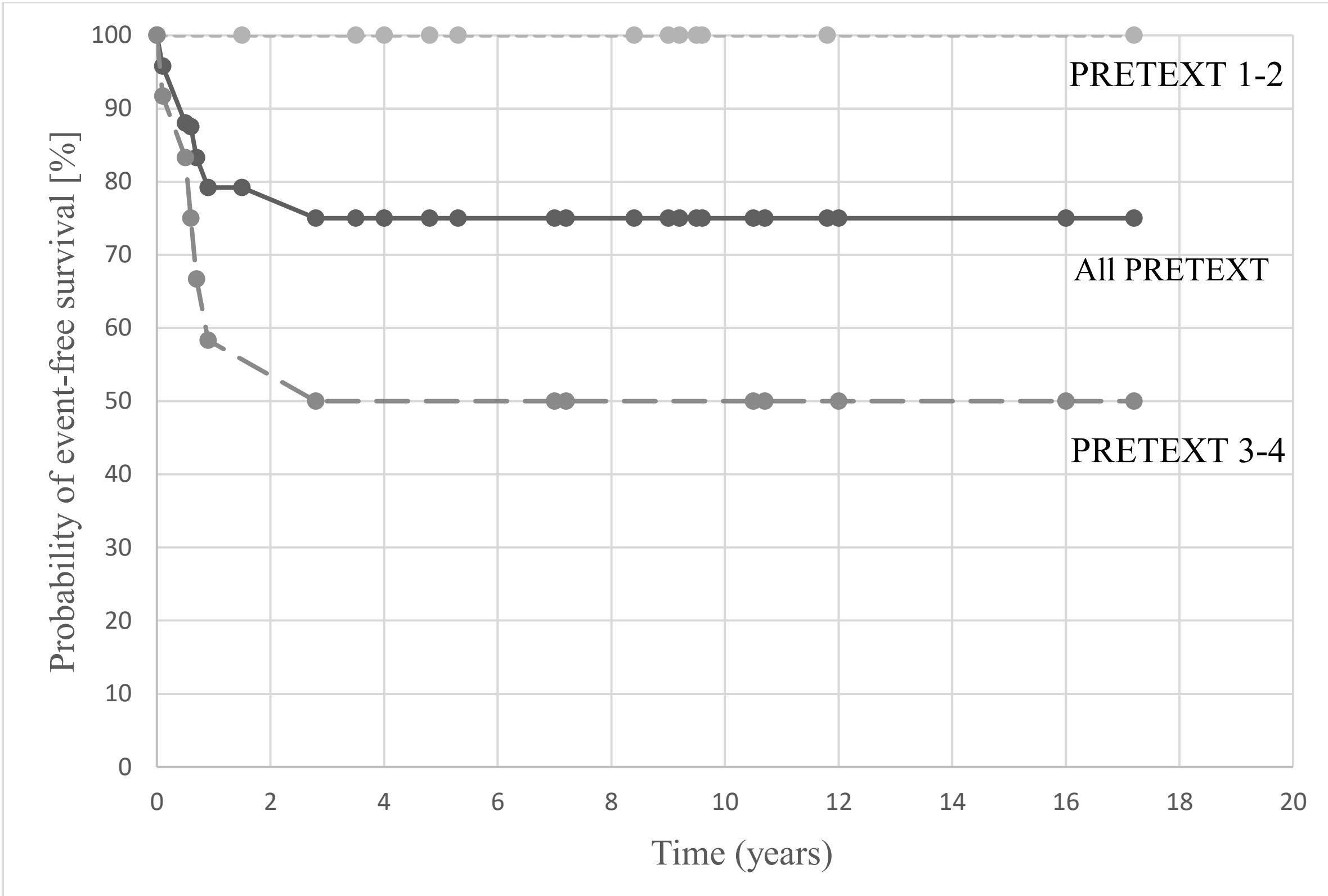

Supplement: Supplementary file 1 [file DataSheet1.pdf]
